# No evidence of Rift Valley fever antibodies in veterinarians and sheep in Northern Palestine

**DOI:** 10.14202/vetworld.2022.1990-1995

**Published:** 2022-08-20

**Authors:** Ibrahim Alzuheir, Belal Abu Helal, Mohammad Abu Helal, Adnan Fayyad, Nasr Jalboush

**Affiliations:** 1 Department of Veterinary Medicine, An-Najah National University, P.O. Box 7 Nablus, Palestine; 2 Department of Public Health Sciences, Faculty of Graduate Studies, An-Najah National University, P.O. Box 7 Nablus, Palestine

**Keywords:** Palestine, Rift Valley fever, seroprevalence, sheep, veterinarian

## Abstract

**Background and Aim:**

Rift Valley fever virus (RVFV) is a vector-borne virus that causes RVF in humans and ruminants. The clinical symptoms in humans and animals are non-specific and often misdiagnosed, but abortions in ruminants and high mortality in young animals are characteristic. Since the initial outbreak in the Rift Valley area in Kenya, the disease has spread to most African countries and the Middle East. The presence and epidemiological status of RVFV in humans and animals in Palestine are unknown. This study aimed to investigate the presence and risk factors for RVF seroprevalence in veterinarians, as occupational hazard professionals, and sheep, as highly susceptible animals, in Northern Palestine.

**Materials and Methods:**

A cross-sectional study was conducted. Data and blood samples of 280 Assaf sheep and 100 veterinarians in close occupational contact with sheep were collected between August and September 2020 using an indirect enzyme-linked immunosorbent assay.

**Results:**

No evidence of RVF antibodies was found in any human or animal sample.

**Conclusion:**

Our results suggest that RVFV has not circulated in livestock in Northern Palestine, yet. Surveillance and response capabilities and cooperation with the nearby endemic regions are recommended. The distribution of competent vectors in Palestine, associated with global climate change and the role of wild animals, might be a possible route for RVF spreading to Palestine from neighboring countries.

## Introduction

Rift Valley fever virus (RVFV) is an emerging arthropod-borne *Phlebovirus* in the family *Phenuiviridae* [1], responsible for causing RVF, a zoonotic infectious disease primarily affecting domestic and wild ruminants, associated with abortions and high mortality rates in young animals [2, 3]. The disease was named after its first discovered incidence: during an outbreak in wool exotic sheep in the Rift Valley area in Kenya [4]. Since then, the disease has been reported in several African countries [5-7].In 2000, the disease emerged in the Arabian Peninsula, causing a significant epizootic/epidemic in Yemen, Saudi Arabia, and Egypt [8-11].

Infection in susceptible animals occurs after the bite of infected mosquitoes (*Aedes* and *Culex* spp.) and possibly those of other blood-sucking insects as well [10, 12]. RVF epidemics are related to heavy rainfall, leading to an increased vector population and the spread of the virus to animals and/or humans [13]. However, the virus needs to be maintained during the inter-epidemic periods by low-level circulation between susceptible hosts and vectors [14, 15]. Transmission to humans occurs through direct contact with infected animal products, such as their blood, bodily fluids, or consuming unpasteurized milk, and less commonly through mosquito bites [16]. Individuals at high risk of RVF infection include veterinarians, laboratory technicians, farmworkers, and workers in the animal processing industry [17]. Veterinarians can serve as sentinels of RVFV outbreaks, even though the virus usually emerges first in animals and then in humans [6]. Besides the aforementioned transmission routes, veterinarians are at a greater risk of infection due to inhalation of aerosols while handling infected animal products, accidental needle-stick inoculation, and injury or broken skin [7]. At present, there is no evidence of person-to-person transmission of RVFV. The clinical disease outcome in humans varies from an asymptomatic, self-limiting, flu-like illness to life-threatening encephalitis, hemorrhagic fever, and death in rare cases [18]. Sheep are highly susceptible to RVF, and the incubation period of the virus is 24–36 h. The mortality rate in adult sheep is 20–30%, while lamb mortality ranges from 95% to 100% [16]. Vertical transmission occurs in all susceptible livestock species, even in pregnant sheep with no detectable viremia [19]. The clinical symptoms are not specific, including subacute death, mild fever, anorexia, or severe hepatitis. The most obvious symptom is the high incidence of abortion in pregnant ewes, which is close to 100% [20].

Considering the public health significance of the disease, its high potential of globalization, the abundance of sheep in Palestine, and the lack of information about the disease, this study aimed to investigate the presence, seroprevalence, and risk factors for RVF in veterinarians, as occupational hazard professionals, and sheep, as highly susceptible animals. These data assist risk assessments and guide decisions on public and livestock health preventive measures.

## Materials and Methods

### Ethical approval and informed consent

The study was approved by the Institutional Review Board of An-Najah National University and the committee of the Faculty of Graduate Studies and Scientific Research Board Council at An-Najah National University with archive number: (5) Nov. 2019. All data regarding participants in this study (veterinarians and animal owners) and the experimental procedures performed were included in the study only after written consent was received from their owners.

### Study period and location

**Figure-1:**
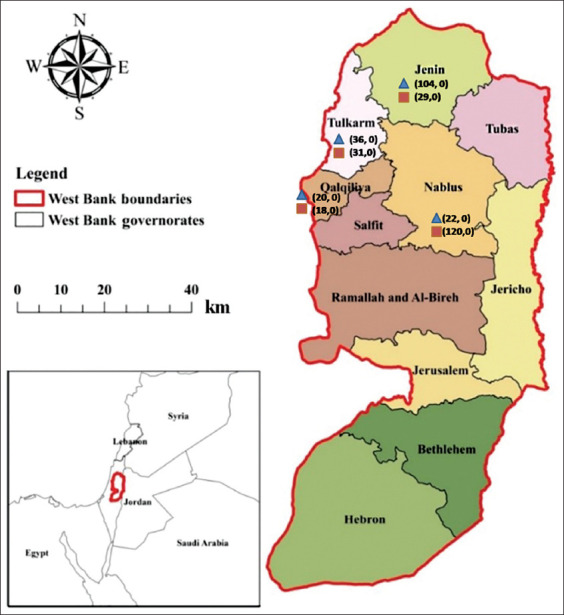
The Map of Palestine (West Bank), showing the areas where the samples were collected. Numbers indicated by a blue triangle and red square for sheep and veterinarians collected and positive samples, respectively. (Source: Map adapted from Shadeed *et al*. [21]).

A cross-sectional study was performed in Northern West Bank-Palestine. The study was conducted from August 2020 to March 2022. Blood samples were collected during August and September 2020 from veterinarians and sheep from five main cities in northern Palestine: Jenin (32°27′40″N 35°18′00″E), Nablus (32°13′20″N 35°15′40″E), Tubas (32°19′20″N 35°22′07″E), Qalqilya (32°11′25″N 34°58′07″E), and Tulkarm (2°18′42″N 35°01′38″E) (Figure-1) [21]. This region encloses an area having close interactions between the vector, wildlife, sheep, and humans. The target area is mainly homogenous, mountainous, and rural. The climate is mainly semi-arid, and the socio-economy is dominantly agropastoral. The climate is semi-desert and represents a transitional zone between the true Mediterranean and desert climate. The distribution and diversification of agricultural patterns include irrigated agriculture in the Jordan Valley to rain-fed farming in the mountains.

### Data and sample collection

A total of 380 samples from veterinarians and sheep from the target areas. The sample size was determined based on methods for estimating a proportion of diseased animals that can precisely represent the entire animal population. With a priori RVF seroprevalence (P) of 50%, maximum allowable error of 5%, and confidence level of 95%, the minimum required sample size was estimated to be 369 samples [22].

### Veterinarians

According to the Veterinarian Association, Jerusalem Branch, the number of working veterinarians in the target areas is 159. Blood samples were collected from 100 veterinarians working in four cities; Jenin (n = 29), Nablus (n = 22), Tulkarm (n = 31), and Qalqilya (n = 18). The veterinarians gave consent to participate in the study and filled out a questionnaire before sample collection. Data regarding age in years, experience in years (1–5, 6–10, 11–15, 16–20, and >20 years), work nature (administrative or field), and work sector (private or public) were collected. Venous blood collection was performed under standard conditions by a trained nurse. The standard blood sample volume was 3–5 mL and was collected using a sterile syringe from a peripheral vein in the antecubital fossa after wiping the region with 70% ethanol. The samples were placed in a plain tube and then transported in an icebox to the Virology Laboratory at the Department of Veterinary Medicine, An-Najah National University. The samples were refrigerated for 2–4 h till the clot fully formed. Serum was obtained by centrifugation at 1006× *g* for 10 min at 25°C and stored at −20°C until future use.

### Sheep

According to the latest data from the Palestinian Central Bureau of Statistics, the estimated sheep population in the target cities is 2,391,169, of which the Assaf breed represents 47.3% (113,127) [23]. All samples were collected from the Assaf breed. The number of animals sampled from each city was: Jenin (n = 104), Nablus (n = 120), Tulkarm (n = 36), and Qalqilya (n = 20) (Figure-1). Data regarding location, age in years (≤1, 1–3, and >3 years), sex, and pregnancy were recorded. After clinical examination, 5 mL of blood was collected from the jugular vein in plain tubes containing a clotting activator (Vacuette, Serum Clot activator, Greiner bio-one, Kremsmunster, Austria) and sent to the Virology Laboratory at the Department of Veterinary Medicine, An-Najah National University for analysis.

### Enzyme-linked immunosorbent assay (ELISA)

Antibodies against the RVFV nucleoprotein were detected using the commercially available ID Screen RVF Competition Multi-species kit IDvet (IDvet Innovative Diagnostics, Montpellier, France). Following the manufacturer’s instructions, serum was considered positive when the sample/positive control ratio (S/P%) was ≥40%. The specificity and the sensitivity of the kit were 100% and 98%, respectively [24].

### Statistical analysis

Categorical variables are described as counts and percentages. The variables for veterinarians were location, age (years), experience (years), work sector, work nature, and type of animals that they interact with. The variables for sheep were location, age (years), sex, and pregnancy. The association between seroprevalence of RVF and the investigated risk factors was determined using the Chi-squared test in the statistical software statistical package for the social sciences (SPSS) version 20 (SPSS Inc., USA). p < 0.05 was considered statistically significant.

## Results and Discussion

RVF is an arboviral transboundary zoonotic disease associated with public and occupational hazards as well as high morbidity and mortality in wildlife and domestic ruminants [2, 3]. In this study; a total of 380 serum samples (100 veterinarians and 280 Assaf sheep) were involved. All samples were found to be seronegative at the sampling time point. No cases of abortion were detected in the investigated animals. Sample variables are shown in Table-1.

**Table-1:** RVF seroprevalence in the sera of veterinarians and sheep in various locations in Palestine by enzyme-linked immunosorbent assay.

Variable	Category	Number of samples (%)	Positive Percentage (%)
Veterinarian			
Location	Jenin	29 (29)	0
	Nablus	22 (22)	0
	Qalqilya	18 (18)	0
	Tulkarm	31 (31)	0
Age (Years)	24–29	40 (40)	0
	30–35	25 (25)	0
	36–40	10 (10)	0
	41–45	5 (5)	0
	>45	20 (20)	0
Experience length (Years)	1–5	37 (37)	0
	6–10	20 (20)	0
	11–15	15 (15)	0
	16–20	8 (8)	0
	>20	20 (20)	0
Sector	Public	34 (34)	0
	Private	66 (66)	0
Work nature	Administrative	11 (11)	0
	Field	89 (89)	0
Animals you interact with	Food animals	49 (49)	0
	Pets	2 (2)	0
	All	49 (49)	0
Sheep			
Location	Jenin	104 (37.1)	0
	Nablus	120 (42.9)	0
	Qalqilya	20 (7.1)	0
	Tulkarm	36 (12.9)	0
Age (Years)	≤g	61 (21.8)	0
	1–3	157 (56.1)	0
	>3	62 (22.1)	0
Sex	Male	63 (22.5)	0
	Female	217 (77.5)	0
Pregnancy	Pregnant	97 (44.7)	0
	Non-pregnant	120 (55.3)	0

The epidemiology of RVF is related to climatic and environmental factors which favor the mosquito population [25]. More than 53 mosquito species have been identified as possible vectors for RVFV [26], including *Phlebotomus papatasi* and *Aedes albopictus* in Palestine [27, 28]. These vectors are related to the endemic circulation of other arboviral diseases in Palestine, like West Nile fever in humans and animals, and bluetongue in ruminants [29, 30]. Controlling such a disease requires a strategy like the One Health approach, which involves understanding and managing the animal, human, and environmental determinants of the disease [10].

Despite the high density of small ruminants, the abundance of competent vectors, and the suitable climatic environment, the sera of the 380 samples (sheep and veterinarians), analyzed by ELISA in this study showed no evidence of RVFV antibodies. This finding aligns with the absence of any official World Organization for Animal Health record of RVFV infection in domestic animals in Palestine; moreover, this disease has never been tested in wild animals [31]. Similarly, the disease has not been reported in the neighboring countries of Jordan and Syria. The absence of RVFV in Palestine is puzzling and might be related to the sampling period in the dry season, when the mosquito vector abundance would be lower than usual, making natural viral circulation less likely. Rare epizootic RVF activity is associated with dry grassland and semi-arid zones found in flood plains [32].

In the last decade, the distribution of RVF has changed significantly [33]. Emerging outbreaks were reported in North Africa [5, 6] and the Middle East [8], which caused a significant epizootic/endemic in 2000. Five RVF epidemics have been reported in Egypt: 1977, 1993, 1994, 1997, and 2003 [9]. Two studies have been reported in Iraq: the first included 1,215 sheep serum samples collected from Basra city in 2012, which revealed an overall seroprevalence of RVFV of 0.89 % [34], and the second included 386 sheep and goat serum samples from Nineveh city, which showed a 2.99% IgG seropositivity rate in October 2012 [35]. These findings indicate that the disease may emerge in these areas. Several studies have developed RVF monitoring and risk mapping with correlative approaches using various environmental measurements and epidemiological data [36, 37]. Understanding the factors responsible for the absence of RVFV could provide valuable information to prevent and control the disease in a region infested by the vector but lacking the virus [25]. Our findings provide a baseline for future prediction and early warning systems for RVFV emergence.< Spatial and temporal surveillance of the vectors and susceptible animals will facilitate early warning [25–32].

## Conclusion

We found no evidence that RVFV has circulated in domestic sheep and veterinarians in Northern Palestine. Samples collected in different seasons and encompassing the whole country would give a better view of the epidemiological status of the disease. The previous reports of the disease in neighboring countries, ongoing climate change, and the abundance of vectors indicate the emergence of RVFV in Palestine. Further studies should include the vectors and wild animals at the national level, as well as collaborations with other North African and Middle Eastern countries. Responsible authorities, namely the Ministry of Health and the Ministry of Agriculture, should strengthen their surveillance and the application of the RVF early prediction system.

## Authors’ Contributions

IA: Designed and planned the study and drafted the manuscript. BAH: Contributed to animals’ blood collection. MAH: Contributed to data and sample collection from the veterinarians. AF: Contributed to data analysis and revised the manuscript. NJ: Contributed to the ELISA work. All authors have read and approved the final manuscript.
